# Supplementary material for: Polarization-independent wavelength demultiplexer based on a single etched diffraction grating device
Source: Commun Eng. 2023 Jan 31;2:4. doi: 10.1038/s44172-023-00055-6 (PMC10955807; doi:10.1038/s44172-023-00055-6)
Supplement: Supplementary file 2 — Supplementary Information [file 44172_2023_55_MOESM2_ESM.pdf]

# Supplementary Information

# Polarization-independent wavelength demultiplexer based on a single etched diffraction grating device

**Chenguang Li, Bo Xiong and Tao Chu***

College of Information Science and Electronic Engineering, Zhejiang University, Hangzhou 310027, China

* Corresponding author. Email: chutao@zju.edu.cn

## Supplementary Note 1:

## Principle of simultaneous demultiplexing of polarization and wavelength

The design of the EDG demultiplexer is based on the principles of blazed grating and Rowland mounting[1,2]. When the difference in the effective optical path is designed as an integer multiple of the wavelength, the diffracted light will be enhanced and light of different wavelengths can be demultiplexed in the EDG. The working principle of a single-polarization EDG is shown in Supplementary Figure 1a, and the grating teeth located at the tangential points of the Rowland circle (green dotted line) and grating circle (black dotted line) are considered as the central grating teeth ($0^{th}$). In the case of single-polarization EDGs (such as TE polarization), the following equations need to be satisfied.

$$(L_{\mathrm{kin}} + L_{\mathrm{kout}}^{\mathrm{TE}}) - (L_{\mathrm{0in}} + L_{\mathrm{0out}}^{\mathrm{TE}}) = \frac{km\lambda}{n_{\mathrm{eff}}^{\mathrm{TE}}}, \tag{1}$$

$$L_{\mathrm{0in}} = L_{\mathrm{f}} \cdot \cos(\theta_{\mathrm{in}}), \tag{2}$$

$$L_{\mathrm{0out}}^{\mathrm{TE}} = L_{\mathrm{f}} \cdot \cos(\theta_{\mathrm{out}}^{\mathrm{TE}}), \tag{3}$$

$$L_{\mathrm{kin}} = L_{\mathrm{f}} \cdot \sqrt{(\sin(\theta_{\mathrm{k}}) + \cos(\theta_{\mathrm{in}}) \cdot \sin(\theta_{\mathrm{in}}))^2 + (\cos(\theta_{\mathrm{k}}) + \sin(\theta_{\mathrm{in}})^2)^2}, \tag{4}$$

$$L_{\mathrm{kout}}^{\mathrm{TE}} = L_{\mathrm{f}} \cdot \sqrt{(\sin(\theta_{\mathrm{k}}) + \cos(\theta_{\mathrm{out}}^{\mathrm{TE}}) \cdot \sin(\theta_{\mathrm{out}}^{\mathrm{TE}}))^2 + (\cos(\theta_{\mathrm{k}}) + \sin(\theta_{\mathrm{out}}^{\mathrm{TE}})^2)^2}, \tag{5}$$

where $L_{\mathrm{f}}$ is the diameter of the Rowland circle[3], $L_{\mathrm{0in}}$ and $L_{\mathrm{0out}}$ are the effective incident and output optical paths of the central grating tooth, respectively, $L_{\mathrm{kin}}$ and $L_{\mathrm{kout}}$ are the effective incident and output optical paths of the $k_{\mathrm{th}}$ grating tooth, respectively, and $\theta_{\mathrm{in}}$ and $\theta_{\mathrm{out}}$ are the incidence and diffraction angles, respectively. $\theta_{\mathrm{k}}$ is the rounding angle of the grating circle corresponding to the midpoint of each grating tooth, $m$ is the diffraction order, $\lambda$ is the incident light wavelength, and $n_{\mathrm{eff}}$ is the effective index of the propagation medium, which is usually considered as the effective refractive index of the fundamental mode of the slab waveguide. Therefore, $\theta_{\mathrm{k}}$ can be obtained using equation (1), and the arrangement of the etched grating can be determined as shown in existing literature[1-4].

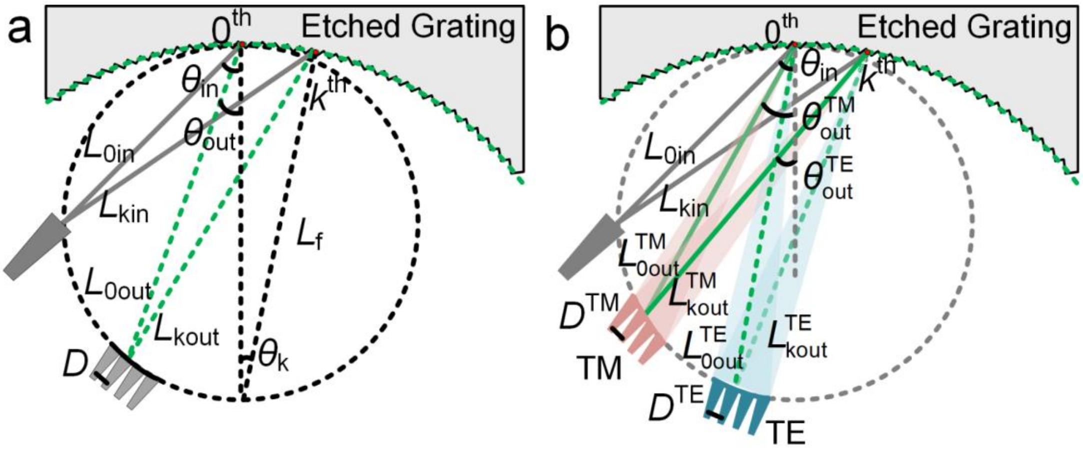


**Supplementary Figure 1. Schematic of EDG design rules. a** EDG design under single polarization. **b** EDG design under TE and TM polarizations.

Further, we utilize the polarization dispersion derived from birefringence to design the polarization-independent EDG, as shown in Supplementary Figure 1b. In the case of TM polarization, a similar phase difference relationship can be considered to realize wavelength demultiplexing.

$$(L_{\mathrm{kin}} + L_{\mathrm{kout}}^{\mathrm{TM}}) - (L_{\mathrm{0in}} + L_{\mathrm{0out}}^{\mathrm{TM}}) = \frac{km\lambda}{n_{\mathrm{eff}}^{\mathrm{TM}}}, \quad (6)$$

$$L_{\mathrm{0out}}^{\mathrm{TM}} = L_{\mathrm{f}} \cdot \cos(\theta_{\mathrm{out}}^{\mathrm{TM}}), \quad (7)$$

$$L_{\mathrm{kout}}^{\mathrm{TM}} = L_{\mathrm{f}} \cdot \sqrt{(\sin(\theta_{\mathrm{k}}) + \cos(\theta_{\mathrm{out}}^{\mathrm{TM}}) \cdot \sin(\theta_{\mathrm{out}}^{\mathrm{TM}}))^2 + (\cos(\theta_{\mathrm{k}}) + \sin(\theta_{\mathrm{out}}^{\mathrm{TM}})^2)^2}, \quad (8)$$

where $L_{0out}^{TM}$ and $L_{kout}^{TM}$ are the output optical paths of the central grating tooth and $k_{\mathrm{th}}$ grating tooth, respectively, in TM polarization. Therefore, the output angle in TM polarization, $\theta_{\mathrm{out}}^{\mathrm{TM}}$, can be calculated by substituting the positions of the grating teeth and effective refractive index of the slab waveguide in TM polarization, and the same diffraction order can be selected for uniform diffractions. Comparing equations (1) and (6), the output angle at different polarizations is dependent only on their refractive indices. Thus, an EDG with simultaneous demultiplexing of polarization and wavelength can be realized.

However, according to equations (6)–(8), selecting different angles $\theta_{\mathrm{k}}$ of the grating teeth may change $\theta_{\mathrm{out}}^{\mathrm{TM}}$, which causes aberrations and increased loss. Therefore, the aberrations of the output under TM polarization should be considered. We calculated the output angles corresponding to each grating tooth of the EDG in both TE and TM polarizations, as shown in Supplementary Figure 2. We can see that the output angles of the grating teeth remain the same in TE polarization. In TM polarization, there are slight changes in the output angles of the grating teeth at the edges, whereas the output angles are almost constant at the central grating teeth, where the light emissions are much stronger. Therefore, we define the output

angles according to the positions of the central grating teeth to obtain minimal aberration and better device performance.

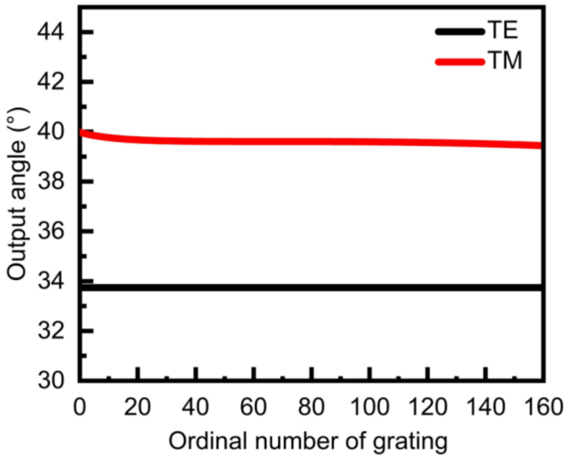


**Supplementary Figure 2. Aberration analysis under TE and TM polarizations.**

# Supplementary Note 2: High-performance EDG design

We optimized the structural parameters of the polarization-independent EDG to improve its performance.

**$Si_3N_4$ thickness**

The thickness of the $Si_3N_4$ layer has a significant influence on the performance of the Bragg gratings and polarization dispersion. The influence of thickness on the performance of the Bragg grating was simulated, as shown in Supplementary Figure 3a. With the thickening of the $Si_3N_4$ layer, the reflection bandwidth of the Bragg gratings increases. To reduce the insertion loss, the reflection bandwidth of the Bragg gratings should cover the wavelength range for demultiplexing in both TE and TM polarizations, which requires that the $Si_3N_4$ thickness $t_{Si3N4}$ should be larger than 250 nm.

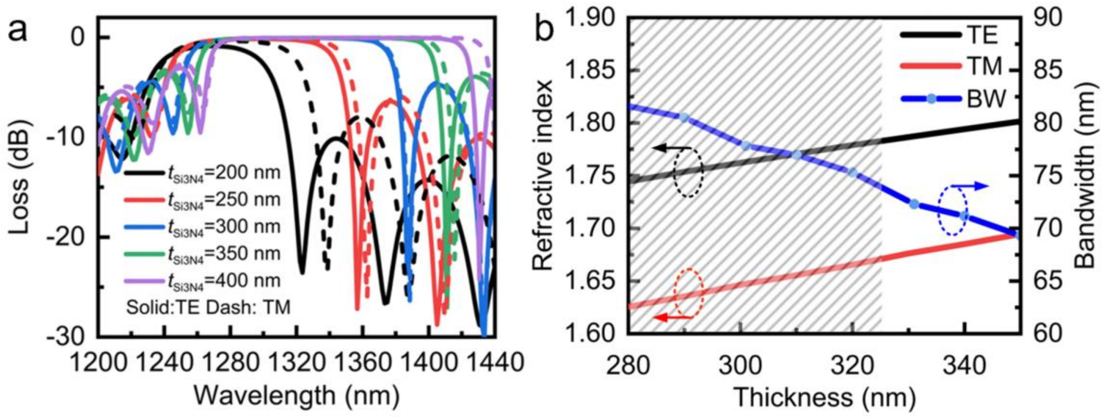


**Supplementary Figure 3. Effect of variation of $Si_3N_4$ thickness on EDG performance. a** Reflection of Bragg gratings at different $Si_3N_4$ thicknesses (period of Bragg grating is 418 nm). **b** Effective refractive indices of TE/TM polarizations and working bandwidth of polarization separation corresponding to the thickness of the $Si_3N_4$ layer at 1310 nm wavelength. The gray area is the thickness for CWDM4 demultiplexing.

The working bandwidths are shown in Supplementary Figure 3b. When the $Si_3N_4$ layer becomes thinner, the difference in $n_{eff}$ between the TE and TM polarizations increases significantly, allowing polarization separation of the EDG outputs over a wider wavelength range. According to the provisions of ITU.G.694.2, $t_{Si3N4}$ should not exceed 325 nm to obtain sufficient separation bandwidth for CWDM4 demultiplexing in O-band. Therefore, we finally selected $t_{Si3N4}$ = 310 nm by weighing the reflection efficiency of the Bragg gratings and working bandwidth of the EDG.

**Diffraction order *m***

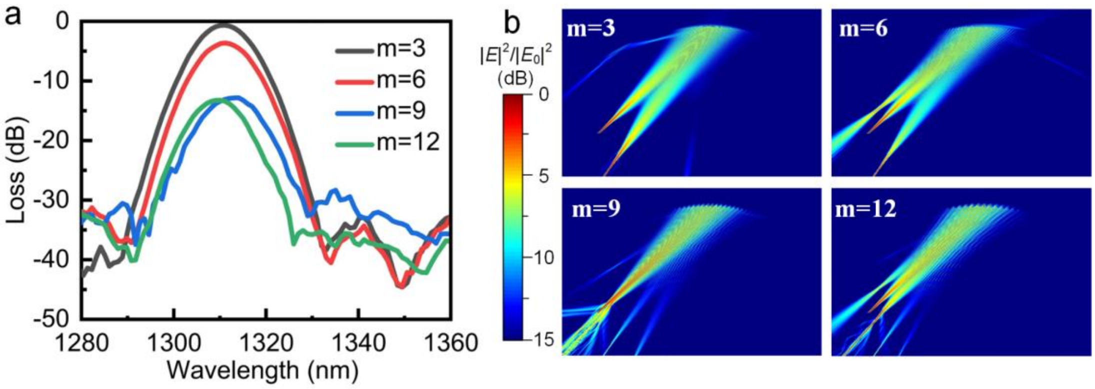


**Supplementary Figure 4. Operating characteristics of EDG at TE polarization for different diffraction orders. a** Output spectra of the central output channel under TE polarization for different diffraction orders. **b** Electric field distribution at $\lambda$ = 1311 nm for different diffraction orders.

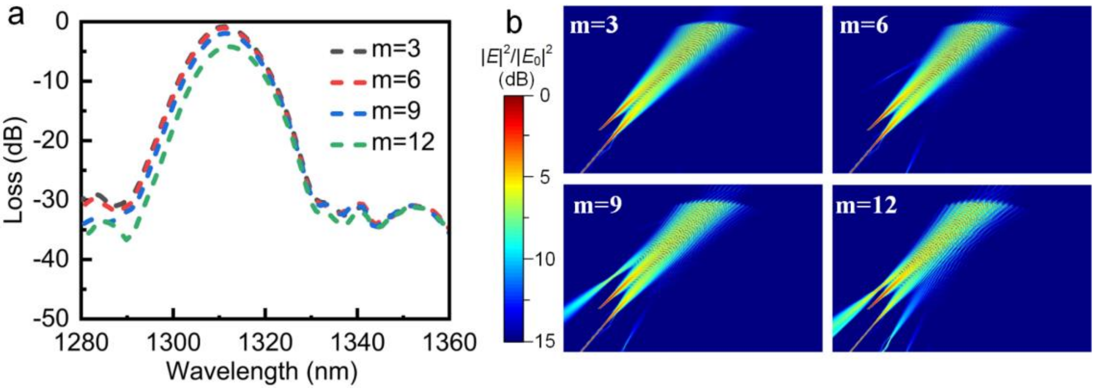


**Supplementary Figure 5. Operating characteristics of EDG at TM polarization for different diffraction orders. a** Output spectra of the central output channel under TM polarization for different diffraction orders. **b** Electric field distributions at $\lambda$ = 1311 nm for different diffraction orders.

As shown in Supplementary Figures 4 and 5, the performances under different diffraction orders were estimated to optimize the diffraction order *m* of the EDG. When *m* gradually increases, the number of grating teeth as well as the free spectral range decreases and causes multiple diffraction orders, which are close to the designed output position. Therefore, when *m* is large, adjacent diffraction orders will absorb the energy of the diffraction order designed by us, resulting in increased loss. As shown in Supplementary Figures 4b and 5b, the EDG exhibits ultra-low insertion losses in both polarizations at *m* = 3. When *m* is larger, the insertion loss of the EDG increases significantly, especially under TE polarization. Moreover, a smaller diffraction order will result in grating teeth that are more compact, which complicates the fabrication process without significant performance improvements. Considering these facts, we selected *m* = 3.

## Output waveguide spacing

Crosstalk is defined as the ratio of the output power at the central wavelength of the channel to the output power at this wavelength of the other channels. As shown in Supplementary Figure 6, to optimize the crosstalk of the EDG, we simulated the output spectra under TE and TM polarizations at 1291 nm (solid line) and 1311 nm (dashed line), respectively, for different values of the output waveguide spacing *d*. As *d* increases, the crosstalk of the device under each polarization shows a decreasing trend. In addition, we noticed that the noise floor under TM polarization is higher than that under TE polarization. To obtain the ideal crosstalk characteristics in both polarizations as well as a compact footprint, $d$ = 5 μm was finally selected as the optimal parameter.

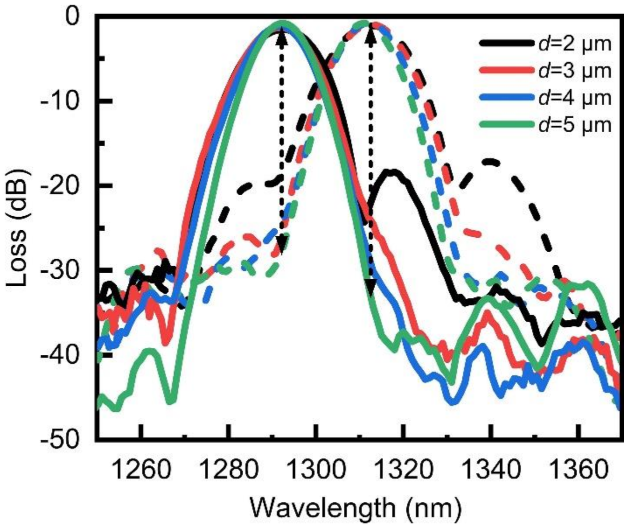


**Supplementary Figure 6. Simulated results of the output spectra of TE (solid line) and TM (dashed line) polarizations at different values of the output waveguide spacing.**

## Supplementary Note 3: Experimental error analysis

During fabrication, the unexpected variation in the thickness of the $Si_3N_4$ layer during film deposition can significantly affect the output wavelengths of the EDG. Therefore, we analyzed the variations in the TE/TM output spectra of the central output channels for different $Si_3N_4$ thicknesses, as shown in Supplementary Figure 7. According to the simulations, an increase of 10 nm in the thickness causes overall red shifts of approximately 6–8 nm of the EDG output spectra in both TE and TM polarizations.

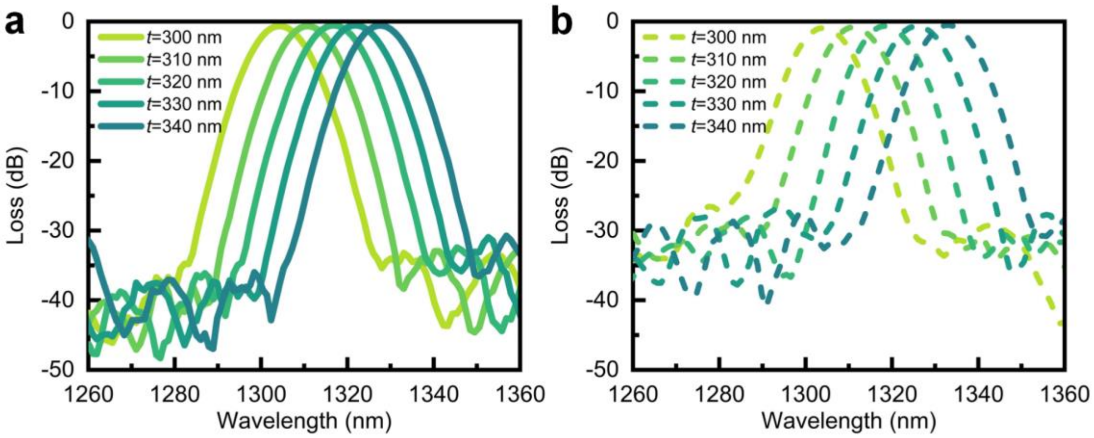


**Supplementary Figure 7. Simulated results of the output spectra for central channel for various $Si_3N_4$ layer thicknesses. a** Transmission spectra of wavelength scans under TE polarization **b** Transmission spectra of wavelength scans under TM polarization

During the actual $Si_3N_4$ deposition process, the thicknesses of the $Si_3N_4$ layer grown in 870 s were 306.5 nm and 325.7 nm owing to the limitation related to the accuracy of the plasma-enhanced chemical vapor deposition equipment. The difference in thickness of 19.2 nm caused a shift in the output wavelengths of the fabricated EDGs. Therefore, the consistency of the output wavelength of the fabricated device can be further improved by using deposition equipment of greater accuracy.

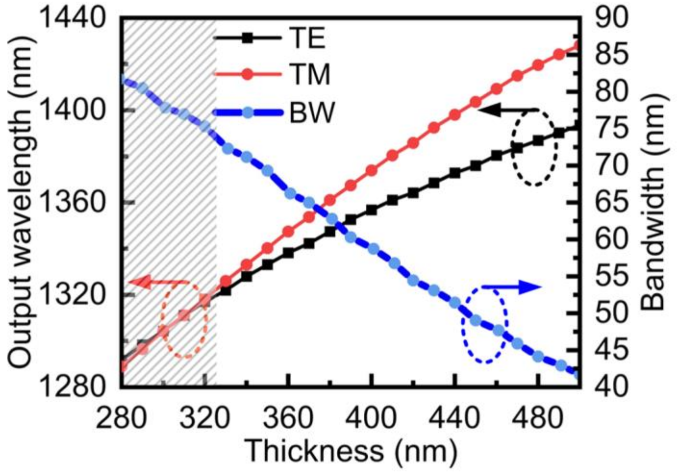


**Supplementary Figure 8. Simulated results of the wavelength shift and polarization dispersion characteristics at different $Si_3N_4$ layer thicknesses.**

Further, we simulated the wavelength shift of the central output waveguide at different $Si_3N_4$ layer thicknesses, as shown in Supplementary Figure 8. As the $Si_3N_4$ layer thickness gradually increases, the wavelength shift sensitivity exhibits a decreasing trend, which means that a thicker $Si_3N_4$ layer can reduce the wavelength shift caused by the thickness change. However, after considering the polarization dispersion performance, the $Si_3N_4$ thickness can only be designed to be less than 325 nm to ensure the required operating bandwidth for CWDM4.

## Supplementary Note 4: Comparison of demultiplexers

**Supplementary Table 1. Comparison of polarization-independent demultiplexing devices.**

| Ref. | Component | Cascaded or not | CMOS compatible | Insertion loss (dB) | PDL (dB) | Crosstalk (dB) | Channel number * spacing | Footprint ($mm^2$) |
|---|---|---|---|---|---|---|---|---|
| **This work** | **EDG (our work)** | **No** | **Yes** | **0.5** | **0.5~1.8** | **-30** | **4*20 nm** | **0.07** |
| [5] | PBS+PR+AWG | Yes | Yes | 6.5 | 2.0 | −12 | 8*0.8 nm | 20 |
| [6] | PSR+AWG | Yes | Yes | 3.6 | 1.2~1.8 | −15 | 8*10 nm | N/A |
| [7] | PBS+PR+ DMZI+AWG | Yes | Yes | 5.5 | 0.5 | −20 | 16*0.8 nm | 4.76 |
| [8] | PBS+AWG | Yes | Yes | 6 | 0.2 | −16 | 8*0.8 nm | ~6.5 |
| [9] | PR+AWG | Yes | Yes | 2.2 | 0.2~0.7 | −29 | 4*20 nm | 1.03 |
| [10] | EDG | No | Yes | 2 | 0.5 | −25 | 40*0.8 nm | 150 |
| [11] | Silica AWG | No | No | 2.5 | N/A | −30 | 4*20 nm | 29.4 |
| [12] | Silica AWG | No | No | 2 | 0.2 | −20 | 8*0.8 nm | ~50 |

The comparison of the device proposed in this paper with other polarization-independent demultiplexing devices reported in recent years is shown in Supplementary Table 1. From this, we can see that our device was superior in terms of both performance parameters such as insertion loss, crosstalk, and PDL, as well as large-scale application requirements such as process compatibility and footprints. Therefore, it is a promising solution for the implementation of SiPh WDM receivers.

## Supplementary References


1. Ye, T. & Chu, T. Low-loss and low-crosstalk Si etched diffraction gratings with multi-point iterative optimization. In *IEEE 13th International Conference on Group IV Photonics* (GFP) (2016).
2. Lycett, R. J., Gallagher, D. F. & Brulis, V. J. Perfect chirped echelle grating wavelength multiplexor: design and optimization. *IEEE Photonics J*. **5**, 2400123–2400123 (2013).
3. Lycett, R. J., Gallagher, D. F. & Brulis, V. J. Perfect chirped echelle grating wavelength multiplexor: design and optimization. *IEEE Photonics J*. **5**, 2400123–2400123 (2013).
4. Song, J., Zhu, N., He, J.-J. & He, S. Etched diffraction grating demultiplexers with large free-spectral range and large grating facets. *IEEE Photonics Technol. Lett.* **18**, 2695–2697 (2006).
5. Chen, L. Doerr, C. R. & Chen, Y. Polarization-diversified DWDM receiver on silicon free of polarization-dependent wavelength shift. In Optical Fiber Communications Conference (OSA) (2012).
6. Zhao, Y. et al. Broadband polarization splitter-rotator and the application in WDM receiver. *IEEE Photonics J.* **11**, 1–10 (2019).
7. Jeong, S.-H. et al. Polarization diversified 16λ demultiplexer based on silicon wire delayed interferometers and arrayed waveguide gratings. *J. Lightwave Technol*. **38**, 2680–2687 (2020).
8. Han, Q., St-Yves, J., Chen, Y., Ménard, M. & Shi, W. Polarization-insensitive silicon nitride arrayed waveguide grating. *Opt. Lett*. **44**, 3976–3979 (2019).

9. Guerber, S. et al. Polarization independent and temperature tolerant AWG based on a silicon nitride platform. *Opt. Lett*. **45**, 6559–6562 (2020).
10. Feng, D., Qian, W., Liang, H., Luff, B. J. & Asghari, M. High-speed receiver technology on the SOI platform. *IEEE J. Sel. Top. Quantum Electron*. **19**, 3800108–3800108 (2013).
11. Liu, L. et al. Low-cost hybrid integrated 4 × 25 GBaud PAM-4 CWDM ROSA with a PLC-based arrayed waveguide grating de-multiplexer. *Photonics Res*. **7**, 722–727 (2019).
12. Ikeda, K. et al. 5.5%-Δ-PLC/silicon photonics hybrid wavelength MUX/DEMUX-and-switch device. In Optical Fiber Communications Conference and Exhibition (OSA) (2021).
